# Care pathway for childhood glaucoma detection and monitoring in Brazil: how advances in primary and tertiary care integration could improve existing barriers

**DOI:** 10.5935/0004-2749.202100102

**Published:** 2021

**Authors:** Carolina P. B. Gracitelli, Christiane Rolim-de-Moura

**Affiliations:** 1 Department of Ophthalmology and Visual Sciences, Escola Paulista de Medicina, Hospital São Paulo, Universidade Federal de São Paulo, São Paulo, SP, Brazil; 2 Centro de Estudos Alcides Hirai, Ver Mais Oftalmologia, Vinhedo, SP, Brazil; 3 Institute of Psychology, Universidade de São Paulo, São Paulo, SP, Brazil; 4 Universidade Federal de São Paulo, São Paulo, SP, Brazil

Childhood glaucoma encompasses a heterogeneous group of diseases characterized by elevated intraocular pressure, which leads to significant ocular changes and, if not treated, can culminate with vision loss in infants and children^(1)^. Although it is an uncommon group of diseases, affecting approximately 300,000 individuals aged <18 years worldwide, it results in blindness in a high percentage of patients^(2)^. Late diagnosis and postponed treatment have negative impacts on the visual prognosis of the disease. Early severe visual impairment is largely known to lead to delayed motor, language, emotional, and cognitive developments^(3)^. Moreover, in low-income countries, the childhood mortality rate can be 60% higher in children with than in children without blindness^(1)^. Despite the onset of prominent and characteristics symptoms (e.g., enlargement of the ocular globe, photophobia, and intense tearing), until recently, late arrival of patients to tertiary referral centers is still common in Brazil.

The many barriers in the field of glaucoma care frequently affect more severely the diagnosis, treatment, and monitoring of children with glaucoma. First, the availability of trained human resources, as much as specific equipment, either for ophthalmological purposes or pediatric anesthetic procedures, for dealing with pediatric glaucoma is a great challenge. This group of diseases is managed in tertiary centers, and these institutions are highly concentrated in urban and economic privileged areas^(4)^. Another significant issue described worldwide is the access barriers related to socioeconomic status. Poor literacy, and other disabilities are important factors that cause parents to delay the treatment of their children and reduce treatment compliance rates^(5)^. Economic issues that impact in health care resources distribution became even more prominent during the coronavirus disease (COVID-19) pandemic in all countries, including Brazil, which lead to new challenges toward childhood glaucoma care. In this context, to reduce human-to-human COVID-19 transmission and maintain assistance, assessment of risks and postponement of nonessential outpatient visits in childhood glaucoma care, are crucial.

To improve these inequalities in eyecare access, the World Health Organization recommended the collection and reporting of information on the met and unmet eyecare needs of the national population that can be extrapolated to childhood glaucoma care. Briefly, recognizing epidemiological data of childhood glaucoma in Brazil should be the first step toward this goal.

In this direction, by improving the evaluation of the Brazilian profile of childhood glaucoma in tertiary centers, we developed a database registry in 2018 that may be used nationwide to describe characteristics of patients across all tertiary centers in the country. A critical step to establish plans for future intervention policies is to better understand the distinctive pattern of childhood glaucoma in each country and thereby define strategies according to their needs. Using a questionnaire, we created a multicenter online database that allows the identification of risk factors and evaluated the outcomes of interventions and treatments^(6)^. Recently, we applied this registry to identify the profile of children with glaucoma who were admitted to the Department of Ophthalmology and Visual Sciences of the Federal University of São Paulo (UNIFESP/EPM), one of the tertiary centers in Brazil that cater to one of the highest numbers of referred cases in this country. In this study, published in 2021, half of the records had not been updated until 2018 to the current diagnostic classification method, which was proposed by the World Glaucoma Association in 2013. The aim of this classification system was to homogenize diagnostic methods for application in epidemiological data collection and research. In this evaluated service, 80% of the patients were male had bilateral disease, and 44% had primary glaucoma, in contrast to those registered in the tertiary centers in high-income countries, where trauma and glaucoma associated with congenital cataract surgery were the most prevalent causes for admission^(7)^. Moreover, this study highlights some limitations of the service. First, some important information was missing from our records (e.g., age at onset symptoms, corneal diameter, and therapy details). Second, although the most common diagnosis in the children was primary congenital glaucoma, the median age at referral center presentation was 4.67 years, what highlights the delayed provision of tertiary care assistance and explains the significant rate of vision impairment and irreversible blindness in this group.

The reasons for referrals to tertiary centers of patients with childhood glaucoma were analyzed by Leite and Rolim-de-Moura^(8)^. Corneal enlargement and its associated symptoms were the principal referral reasons for patients with primary congenital glaucoma. However, in 97% of the cases, the patients’ parents or caregivers detected the initial symptoms, not health-care professionals. In this regard, we suggest the necessity to increase awareness about this group of diseases in childhood primary care. Social media can play an important role in disseminating education through many different platforms such as educational videos to the primary care chain, which involves pediatricians and pediatric ophthalmologists in our case. Moreover, Leite and Rolim-de-Moura found that in >50% of patients referred to tertiary centers, glaucoma was ruled out. Of the 94 children referred from 2012 to 2018 because of suspected optic disc appearance (increased cup-to-disc ratio or cup-to-disc ratio asymmetry), only eight (8.5%) had a confirmed final diagnosis of glaucoma. This finding increases the attention to the necessity of delivering information and ophthalmological structures such as new examination protocols to primary care ophthalmologists to not only conserve the resources of tertiary care centers but also avoid unnecessary travel of patients’ families.

Unfortunately, no perfect and widespread pathway has been established for glaucoma care that adequately integrates primary to tertiary centers in other health-care systems. However, such challenges must be addressed^(9)^. Recently, representative societies engaged themselves (Brazilian Glaucoma Society, Brazilian Pediatric Glaucoma Society, Brazilian Pediatric Ophthalmology Society, and Brazilian Council of Ophthalmology) in a survey and found 47 ophthalmic services widespread in 19 states in the country, in which assistance was being provided by the Sistema Unico de Saúde. All these services were analyzed and found to possess trained human resources with technological equipment to assist and treat children with glaucoma from the first days of life. Specialists in these centers are now motivated to initiate a more collaborative approach and help develop policies directed toward referral systems to deal with the complex group of diseases described herein. To improve the affordability and effectiveness of health care, radically significant changes in attitudes and behavior are required. In the context of Brazil, training for the primary care setting, which is composed of pediatricians and pediatric ophthalmologists, and integration of a correct referral system to decentralize arrivals at tertiary centers with a proper infrastructure and trained specialists are needed for the early treatment of the diseases. In other words, although we are still at the beginning of a complex process, early detection, prompt referral, specialized team care close to home by tertiary centers must be offered. In addition, the possibility to return to primary centers for correction prescription and close follow-up are strategies that shall improve visual habilitation.

In conclusion, childhood glaucoma remains an ever-challenging disease to manage. Especially after the COVID-19 pandemic, traditional care pathways for detection and monitoring became even more scarce. However, communication tools available during this period allowed the identification of leaders and tertiary care centers, thereby providing opportunities to educate pediatricians and specialized ophthalmologists about the disease. Certainly, this historic moment may be seen as a portal that will prepare the society for engaging in a more equitable world. Appropriate and early treatment remains the cornerstone of the outcome of childhood glaucoma. Instead of wasting time in solving the problems of hyperinflated services, appropriate and early treatment remains the cornerstone of the outcome of childhood glaucoma. Instead of wasting time in solving the problems of hyperin flated services, adequate policies should be developed to facilitate primary care centers integration to descentralized tertiary care centers.
